# Sustainable production of high strength fiber reinforced mortars using volcanic ash and magnetized water treatment technology

**DOI:** 10.1038/s41598-025-06883-0

**Published:** 2025-07-01

**Authors:** Mostafa M. Keshta, Mohamed M. Yousry Elshikh, Osama Youssf

**Affiliations:** Structural Engineering Department, Mansoura University, Mansoura, Egypt

**Keywords:** HS-FRCM, HS-FRGM, Volcanic ash, Magnetized water, Durability, Curing methods, Structural materials, Engineering, Civil engineering

## Abstract

In recent decades, concerns about the high cement consumption and its associated carbon footprint have prompted significant efforts in the construction sector to incorporate alternative materials into cementitious composites. This study focused on the development of a sustainable approach to produce high strength fiber reinforced cementitious mortar (HS-FRCM) and high strength fiber reinforced geopolymer mortar (HS-FRGM). The proposed mortars incorporate volcanic ash (VA) as partial replacements for conventional components, with substitution levels of up to 80%. Furthermore, magnetized water (MW) was utilized as the mixing water in producing both HS-FRCM and HS-FRGM, replacing tap water (TW) for sustainable mortars. Four different curing conditions were used; tap water, seawater, air, and sunlight. The slump values, mechanical performance, durability, and microstructural were conducted and analyzed. The results indicated that VA significantly enhanced HS-FRCM workability by up to 150%, while it had a less pronounced effect on HS-FRGM workability. When 20% VA was used, the 28-day compressive strength of HS-FRCM was not affected, but the compressive strength of HS-FRGM decreased by only 6%. The highest compressive strength was recorded for both HS-FRCM and HS-FRGM when cured in tap water, compared to other conditions of curing. Utilizing MW improved HS-FRCM and HS-FRGM workability by up to 100%, and the compressive strengths increased by as much as 15%. The microstructural analyses revealed that the use of MW resulted in a denser structure with a stronger bond between the fibers and the matrix, as well as fewer microcracks and pores, compared to mixtures prepared with TW. Fourier-transform infrared (FTIR) spectroscopy indicated the effectiveness of using VA and MW in enhancing hydration process.

## Introduction

One of the main reasons of the world’s greenhouse gas emissions is the cement production^1^. When limestone, clay, and other materials are heated to high temperatures for the purpose of making cement, a significant amount of carbon dioxide (CO_2_) is released into the environment. The United States Geological Survey (USGS) estimates that around 4.1 billion metric tons of cement were produced worldwide in 2020^2^. It is estimated that the cement industry produces 7% of the world’s CO_2_ emissions^3^. However, the environmental effects of the production of cement go beyond its role in contributing to greenhouse gas emissions. It also releases other pollutants such as particulate matter, sulfur dioxide (SO_2_), and nitrogen oxides (NOx). In proximity, these pollutants may cause respiratory disorders and other health concerns in local communities^4^. Utilizing alternative raw materials, improving energy efficiency, and putting carbon capture and storage technologies into practice are some of the steps taken to reduce the negative environmental effects of cement production^5,6^. One of these alternative raw materials is the volcanic ash (VA) which is considered one of the natural pozzolanic materials^7^.

VA is a pozzolanic material produced within volcanic eruptions^8^. It is an amorphous, finely grained material made up of glassy particles smaller than 2 mm in diameter^9^. The type of volcano and the location of the eruption affect its chemical composition. But in general, it has significant amounts of iron oxides, aluminum, and silica^10,11^. In general, the use of VA as a sustainable binder in concrete has recently attracted a lot of interest^12^. According to Celik et al.^13^, when VA was used as a replacement of cement at mass percentages of 30% and 50%, the water demand increased. When VA replaced cement by up to 20% in another investigation, it was found that the workability of the concrete increased^11^. According to Mohamad et al.^12^, employing VA at a 15% substitution level can extend the curing duration and raise the cement mortar’s compressive strength. Another study used 20% VA by mass of cement to produce high-performance concrete with a compressive strength of 60 MPa^14^. In the process of producing a VA-based geopolymer, Djobo et al.^15^ largely substituted calcined oyster shell and bauxite for VA. They observed a reduction in the initial setting time (calcined oyster shell) and a favorable effect on the mechanical characteristics of the geopolymer specimens. Bondar et al.^16^ observed a reduction in compressive strength and a percentage of expansion in prisms length of up to 19.4% and 0.074, respectively, in their study investigated the sulfate resistance of alkali-activated andesite powder-based geopolymer concrete. According to Melya^17^, employing the VA can enhance concrete’s tensile and compressive strengths by 5.9% and 9.8%, respectively. Rahmi et al.^18^ demonstrated that when the ratio of VA to cement increased, the workability of the concrete decreased.

The incorporation of different fibers types to improve the performance of mortar or concrete is a subject of considerable interest. Glass fibers (GF), in particular, play several roles in concrete, such as controlling cracks, inhibiting their propagation, and modifying the behavior of materials by controlling the cracks. According to Mirza and Soroushian^19^, adding 1–2% GF to cement-based materials effectively reduces shrinkage cracks, increases toughness, and enhances resistance of temperature in lightweight concrete. Iskender and Karasu^20^ reported that while the inclusion of GF hasn’t any effect on the concrete modulus of elasticity, it enhances the stress–strain behavior and improves flexural strength.

Geopolymer mortar, as a novel construction material, has increasingly emerged as a viable alternative to traditional cement mortar because of its low carbon dioxide emissions and excellent environmental compatibility^21^. It demonstrates significant potential for use across diverse sectors, including construction, civil engineering, and transportation^22^. Recent studies primarily employ bulk solid waste materials like fly ash (FA), ground granulated blast-furnace slag (GGBFS), and silica fume as precursors in geopolymer concrete or mortar as a fully replacement of cement, while the alkaline activators are typically based on sodium hydroxide (SH) solution, sodium silicate (SS) solution, or a combination of the two^23^. At present, researchers have successfully produced high-strength geopolymer mortar, achieving compressive by using FA with a range of precursor materials^24^.

In recent decades, there has been much research on using magnetized water (MW) as a mixing water for concrete. MW is a regular tap water (TW) that has had its characteristics altered by an electric or permanent magnetic field^25^. The molecules in water increase as a result of water magnetization, which lowers its surface tension and increases the amount of dissolved inorganic ions in the water^26^. During the cement hydration reaction, MW easily penetrates the core region of cement particles, increasing its effectiveness and producing cement hydration products with higher quality and density^27^. Su and Wu^28^ studied the workability and compressive strength of mortar containing GGBFS and MW. According to the results, using MW enhanced the mortar’s workability by up to 9% and the compressive strength by up to 19%. Yousry et al.^29^ examined how applying MW could increase cementitious mortar’s compressive strength and found that water circulating for 150 cycles achieved the best compressive strength without FA. The number of cycles the water underwent in magnetization with the proportion of FA were shown to control the increase in compressive strength. When water was magnetized for 150 cycles, the best outcomes were observed. Utilizing MW at a magnetic field intensity of 1.4 tesla, ELShami et al.^30^ discovered how to enhance the hydration products for self-compacting concrete. This study discovered that MW enhanced the workability by up to 11% over that of TW. The MW mixtures outperformed the control mix in terms of flexural, splitting, and compressive strengths at all ages. According to Keshta et al.^31^, when MW was used, the compressive strength of sustainable concrete containing 5% VA rose by up to 12%. Ahmed et al.^32^ examined the effects of the mixing-water magnetization method on the performance of silica fume concrete. They found that the best results for the mechanical properties of the concrete were obtained when the water was magnetized for 150 cycles in 1.4 T and 1.6 T magnetic fields. The compressive strength was improved by up to 80%, the splitting tensile strength by up to 98%, and the flexural strength by up to 22% when MW and silica fume were used. The characteristics of sustainable concrete containing metakaolin and MW were examined by Elkerany et al.^33^. Using TW, this study discovered that the slump of concrete could be reduced by 8% by adding more metakaolin to the mixture, but employing MW increased the concrete slump by 3%.

As per the above literature review, rare research has been conducted in combining VA and MW to produce HS-FRCM and HS-FRGM. This research studied the effect of using both VA and MW on the mechanical, durability, and microstructure properties of HS-FRCM and how they were subsequently developed into the corresponding HS-FRGM. The performance of the mortars was evaluated by measuring the compressive strength in different curing environments, as well as the uniaxial tensile strength and flexural strength. Additionally, the absorption rate and compressive strength were also determined under freezing and thawing conditions and at high elevated temperatures. Microstructure analyses including scanning electronic microscope (SEM), mapping analysis, and Fourier transform infrared (FTIR) were also carried out to investigate the morphology surface of the proposed HS-FRCM and HS-FRGM mixtures. This research is conducted within the framework of ongoing investigations into the use of MW for producing high-strength composites, including engineered cementitious composites (ECC) and engineered geopolymer composites (EGC).

## Materials and methods

### Raw materials

The raw materials used in this investigation to produce the HS-FRCM and HS-FRGM included Portland cement (PC-52.5), river sand, ultra-fine GGBGS, FA, VA, river sand, superplasticizer (SP), SS, SH, TW, and MW, and GF. The solutions of SS and SH were blended to produce the alkaline activator (AA) required for the HS-FRGM. The specific gravities of GGBFS, FA, PC, and VA were 2.83, 2.57, 3.15, and 2.68, respectively. These cementitious materials were sieved through a 170-mesh to ensure they were fine enough for proper mixing, hydration, compaction, and quality control. Chemical composition of these materials is shown in Table 1. The river sand used had a size range of 0.16/4.75 mm with a 2.63 as a specific gravity. The SP was a high range water reducer (type G) with a 1.08 as a specific gravity. Table 2 presents the characteristics of the GF utilized in this study.

**Fig. 1:**
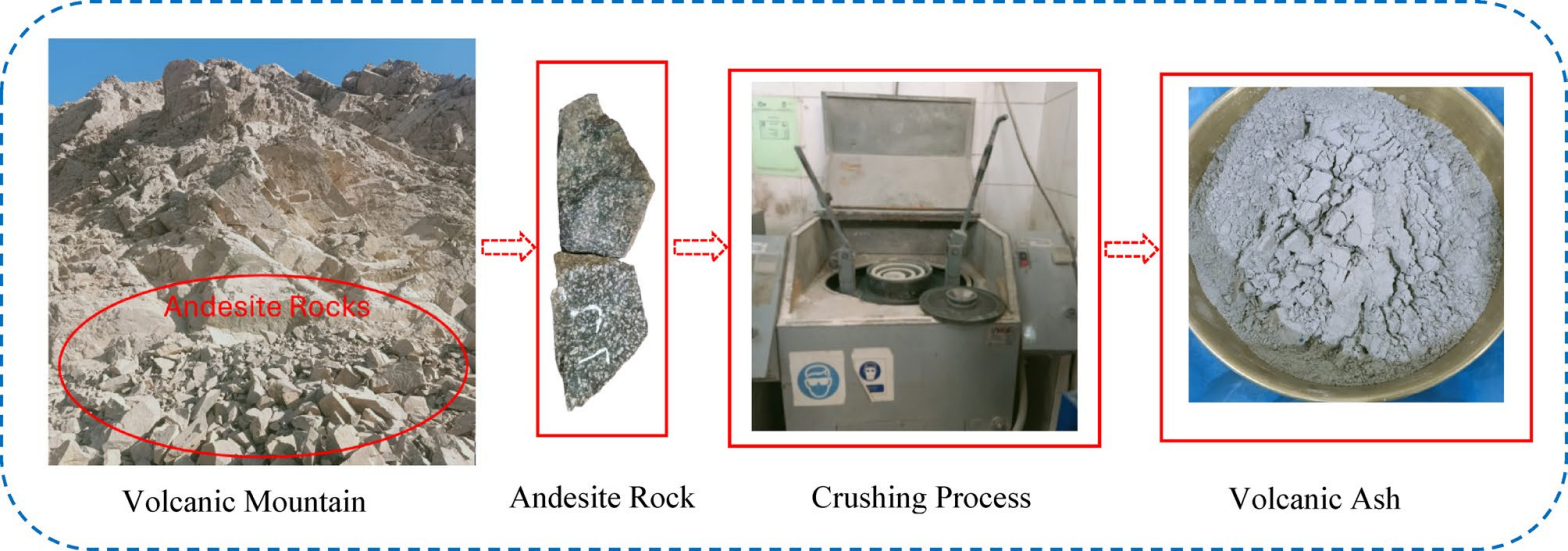
Process of producing VA.

Based on the data sheet, the SS solution consists of 29.4% SiO_2_, and 14.7% Na_2_O, 55.9% water. A 12 M (molarity) SH solution was used, which was prepared by dissolving 400 g (equivalent to 12 times the molecular weight) of solid particles SH in 1 L of water for 5 min. The solid SH, available in pellet form, had a molecular weight of 40 g/mol and a purity level of 98%. It was mixed with SS in a weight ratio of 2:1. The VA was a natural pozzolanic material. Its production involves a series of steps starting by collecting the volcanic rock from its source in Egypt. These rocks were then crushed into VA particles using a crushing machine, as illustrated in Fig. 1.

**Table 1:** Chemical elements of materials used.

Element	SiO_2_	Al_2_O_3_	CaO	Fe_2_O_3_	MgO	SO_3_	Na_2_O	LOI	K_2_O	TiO_2_
PC	20.2	6.0	62.7	3.3	2	2.2	0.01	1.7	----	----
GGBFS	32.8	13.4	43.1	0.4	5.5	1.9	0.40	0.8	0.3	----
FA	51.1	18.1	5.8	9.7	7.3	1.0	3.94	0.20	1.84	0.8
VA	65.7	13.90	3.38	6.12	1.35	0.15	3.95	0.70	3.35	0.80

**Table 2:** Properties of GF fiber used in this study.

Diameter (µm)	Length (mm)	Specific weight (g/m^3^)	Elastic modulus (GPa)	Tensile strength (MPa)
13	6	0.91	72	480

**Fig. 2:**
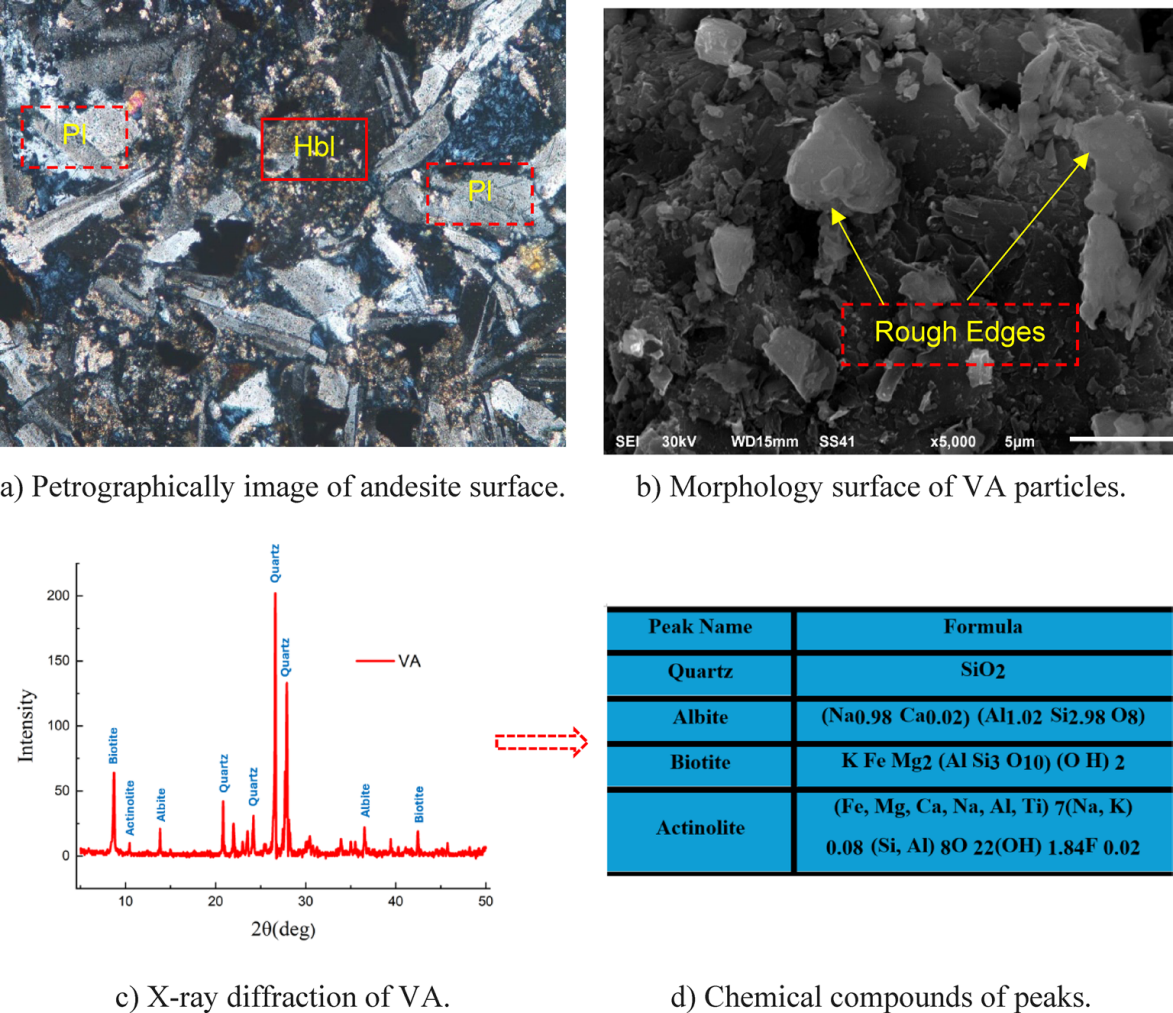
Microstructure analysis of VA particles.

The morphology surfaces of the andesite rocks and VA particles are shown in Fig. 2a. A thin section was prepared to analyze the rock’s composition. The image reveals that the rock colors range from greyish green to greenish gray. Plagioclase (PL) and hornblende (Hb) are the main components of andesite rocks. These minerals, which include chlorite and actinolite, as well as opaque oxides, display porphyritic textures. Plagioclase appears as tabular or prismatic phenocrysts or in the groundmass as fine-grained subhedral laths (up to 1.4 mm long). The tabular euhedral to subhedral plagioclase phenocrysts have slightly altered cores. Macroscopically, andesite rocks are fine-grained, large, and dark grey to green, with a porphyritic texture. Andesite is primarily made up of plagioclase with a few hornblende phenocrysts embedded in a fine-grained groundmass of plagioclase laths, chlorite, opaque oxides, and calcite in thin section. Previous studies^34,35^ indicated that the rock used has a positive environmental impact and is a good alternative mineral material for use in HS-FRCM and HS-FRGM. Figure 2b shows the morphology surface of VA particles using SEM analysis to investigate the shape and the distribution of these particles. Figure 2c and d show X-ray diffraction (XRD) of VA particles and confirm the existence of SiO_2_ in the upper peak point.

### Magnetized water production

**Fig. 3:**
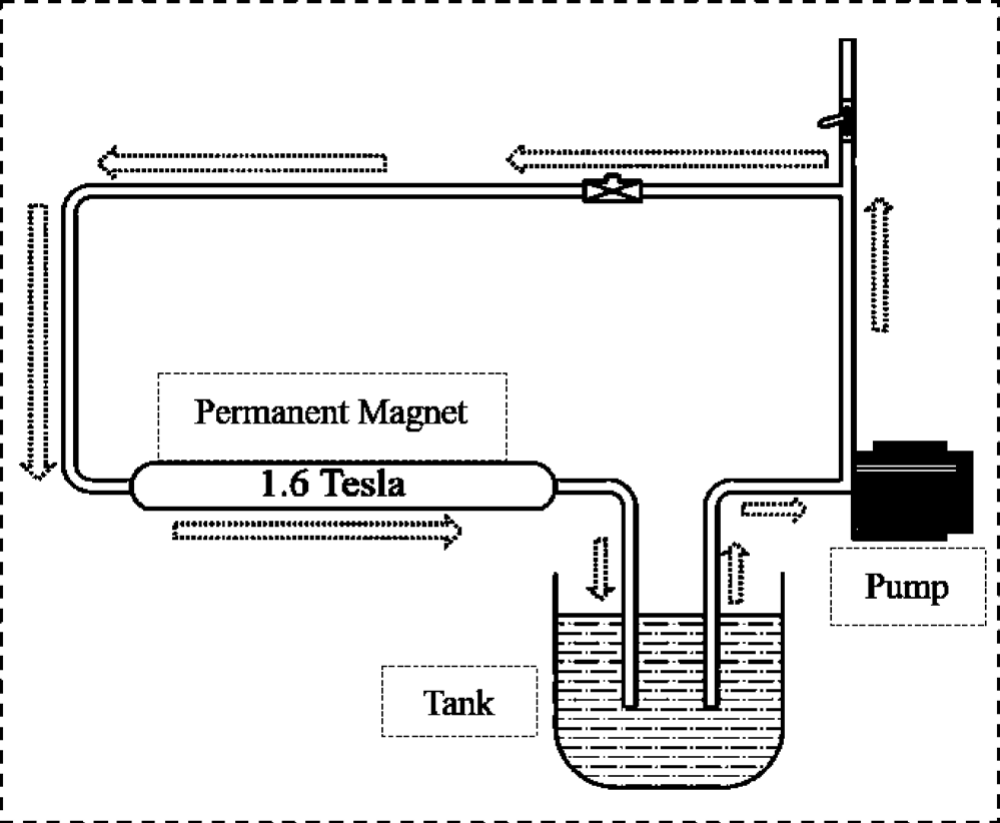
Schematic drawing for setup used in water magnetization^36^.

In this study, two different forms of mixing water were used: TW and MW. MW was produced by exposing TW to a magnetic field with a strength of 1.6 Tesla for 150 cycles. The number of cycles was chosen according to recommendations from previous studies^7,29^, which suggested it as the ideal number for improving the general performance of concrete. Figure 3 shows the setup utilized for water magnetization in this study. The setup contained a permanent magnet with a magnetic intensity of 1.6 Tesla, a water tank, a pump for water pumping, and a group of connected pipes equipped with some valves to regulate water flow. In each magnetization cycle, TW was circulated by pumping it from the tank through the pipes, passing it through the magnetic field, and then returning it to the tank.

### Mixes and variables

This experimental investigation involved 14 mixes divided into two groups: one group for HS-FRCM mixes and the other group for HS-FRGM mixes. Identical parameters were applied to both groups to confirm a valid comparison between the behaviors of HS-FRCM and HS-FRGM. VA was used as a partial replacement of cementitious materials (PC and GGBFS equally in the HS-FRCM group, and FA and GGBFS equally in the HS-FRGM group) at 20%, 40%, 60%, and 80% by volume. Also, the impact of MW on the control HS-FRCM/HS-FRGM mixes and those have 20% VA was conducted. The other mix components in each group were remained constant. Furthermore, Table 3. shows the mix components for two groups. It is worth noting that in VGMW and VG20MW mixes (see Table 3. HS-FRGM group), the MW was used to prepare the SH solution. The fiber, sand, SP, and water contents were maintained consistently across all mixtures at 3% (of mix volume), 571 kg/m³, 1.2% (of binder weight), and 330 kg, respectively.

**Table 3.** Proportions of mixes (kg/m^3^).

**Group name**	**Mix ID**	**Sand**	**PC**	**GGBFS**	**FA**	**VA**	**GF**	**SP**	**Water**	**AA**	
										**SS**	**SH**
**HS-FRCM**	VC	571	643	577	--	0	27.3	14.8	330	--	--
	VC20	571	514	462	--	219	27.3	14.8	330	--	--
	VC40	571	385	346	--	437	27.3	14.8	330	--	--
	VC60	571	257	231	--	656	27.3	14.8	330	--	--
	VC80	571	128	115	--	875	27.3	14.8	330	--	--
	VC-MW	571	643	577	--	0	27.3	14.8	330	--	--
	VC-20MW	571	514	462	--	219	27.3	14.8	330	--	--
**HS-FRGM**	VG	571	--	577	524	0	27.3	--	--	293	147
	VG20	571	--	462	419	219	27.3	--	--	293	147
	VG40	571	--	346	315	437	27.3	--	--	293	147
	VG60	571	--	231	210	656	27.3	--	--	293	147
	VG80	571	--	115	105	875	27.3	--	--	293	147
	VGMW	571	--	577	524	0	27.3	--	--	293	147
	VG20MW	571	--	462	419	219	27.3	--	--	293	147

The HS-FRCM blending process involved several specific steps: At the start, half of the mixing water and half of the SP were added to the mixer. Subsequently, the pre-mixed dry components (GGBFS, PC, VA, and sand) were gradually introduced, followed by the addition of the remaining water and SP. The entire mixture was then mixed for 4 min. Subsequently, GF was gradually introduced as mixing continued. Once all the fibers had been incorporated, mixing proceeded for an additional 5 min to confirm thorough dispersion of the fibers throughout the cementitious matrix. The freshly prepared HS-FRCM was then poured into moulds and covered with plastic wrap. All specimens were demolded after 1 day, and curing began using either tap water, seawater, air, or sunlight, continuing until the testing day. For HS-FRGM mixing, the same procedures were followed as for HS-FRCM, with the distinction that FA in HS-FRGM corresponded to PC in HS-FRCM, and AA in HS-FRGM corresponded to water and SP in HS-FRCM.

### Tests methods

#### Slump test and mechanical performance

**Fig. 4:**
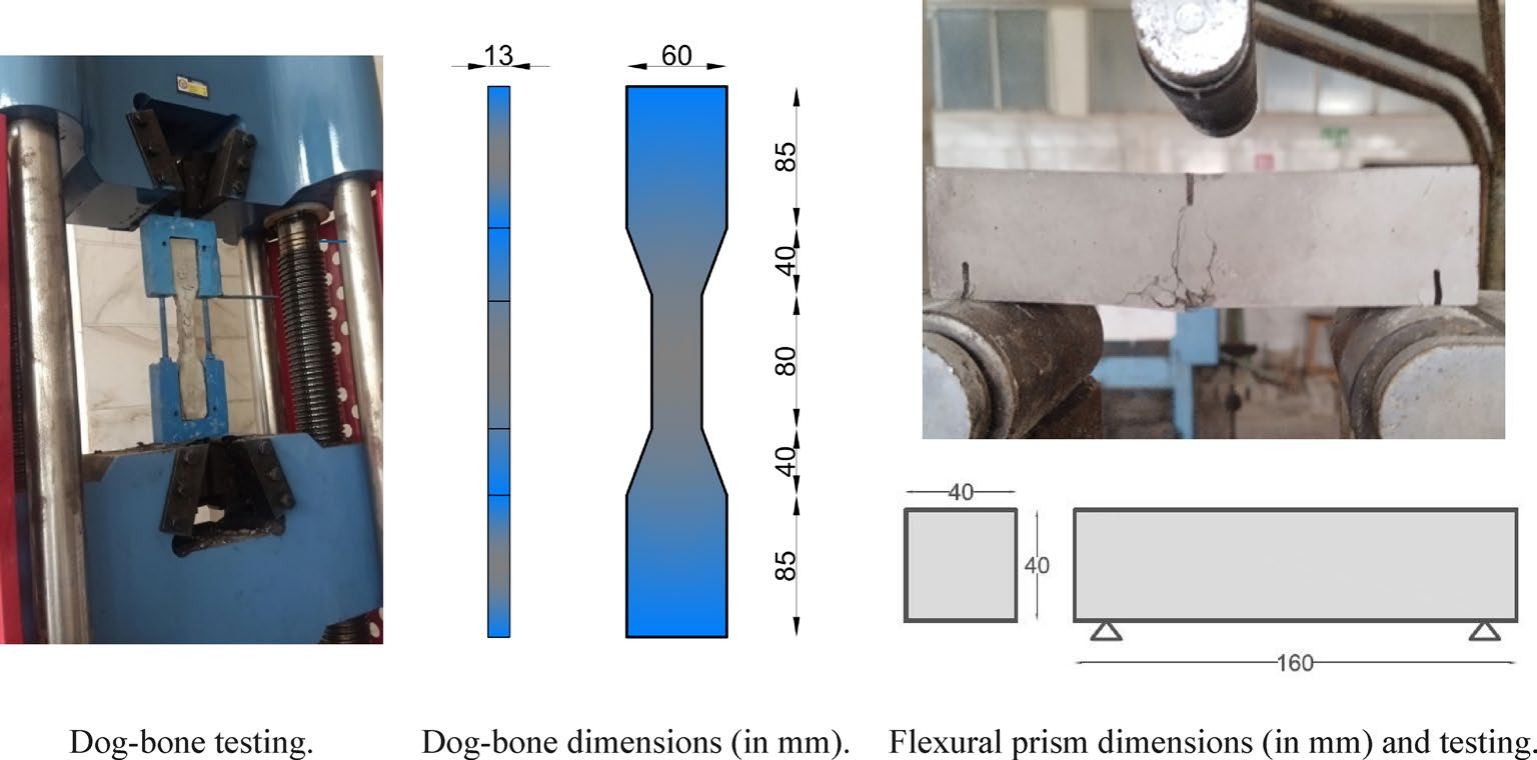
Uniaxial tensile and flexural testing of HS-FRCM and HS-FRGM.

The slump values of the HS-FRCM and HS-FRGM mixtures were evaluated using the slump cone test, conducted with a standard slump cone. The mold had a bottom diameter of 200 mm, a top diameter of 100 mm, and a height of 300 mm, in accordance with ASTM C143-10^37^. The compressive strength of the mixtures was determined by testing 50 mm cubes at 7, 28, and 90 days. A total of 18 cubes were tested for each mix: 3 cubes at 7 days, 12 cubes at 28 days (3 cubes for each of the four curing methods), and 3 cubes at 90 days, following the ASTM C109^38^. The compressive strength value of the mixtures was determined by calculating the mean value of three test specimens. The tensile strength of the HS-FRCM and HS-FRGM mixtures was measured using a uniaxial tensile test conducted in accordance with the JSCE standard^39^. Dog-bone shaped specimens (2 per mixture) were used for this test at 28 days of tap water curing. These specimens were designed to have a smaller cross-section within the gauge length to promote micro-cracking, ensuring a stable setup and accurate measurement of tensile deformation. The dog-bone specimens featured a gauge length of 80 mm and cross-sectional dimensions of 30 mm in width and 13 mm in thickness, as illustrated in Fig. 4. The flexural strength was evaluated using three prism samples measuring 40 mm × 40 mm × 160 mm at 28 days of water curing, by conducting a three-point bending test according to ASTM C348-14^40^, as shown in Fig. 4.

#### Durability evaluation

**Fig. 5:**
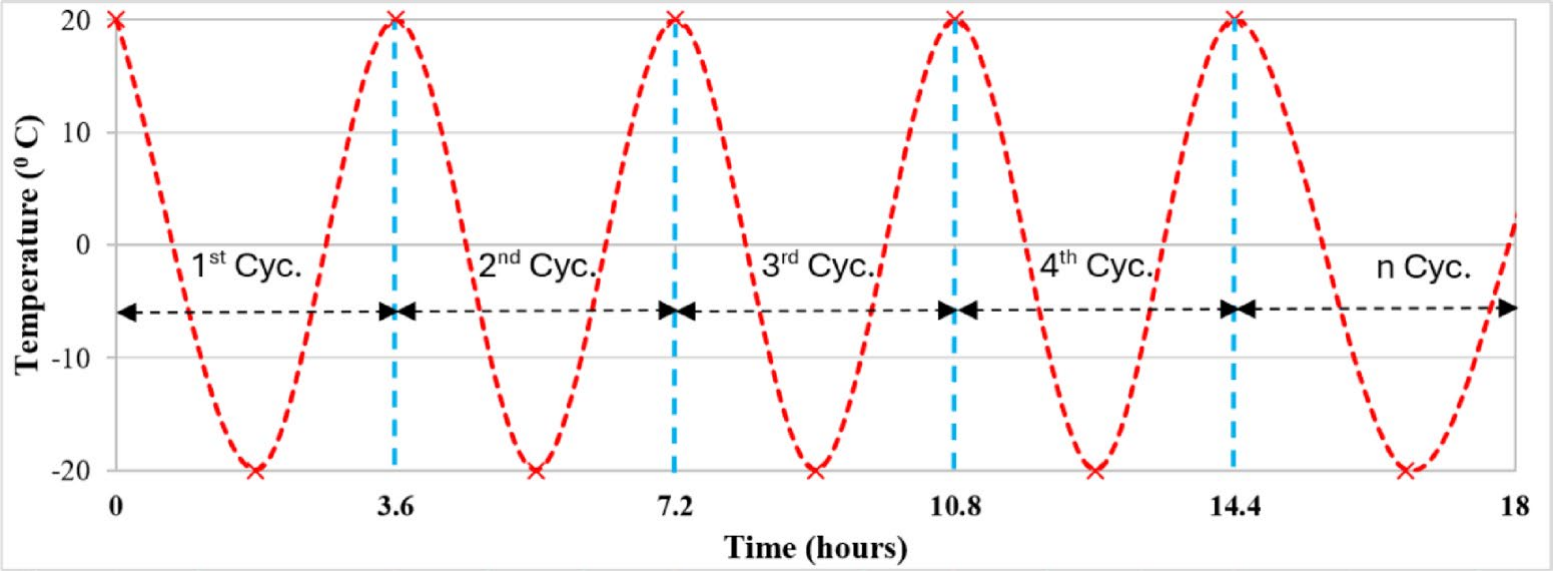
Setup of the rapid freeze-thaw cycles test.

According to the Chinese standard GB/T50082-2009^41^, the rapid freeze-thaw cycle test was conducted on 28-day hardened HS-FRCM/HS-FRGM specimens (50 mm cubes) using traditional freezer. Figure 5 illustrates the temperature change for the rapid freeze-thaw cycle test in this study. As shown in the figure, each freeze-thaw cycle took about 3.6 h^42^. During the freeze-thaw cycles, the temperature ranged between − 20 °C and 20 °C for 300 cycles. Prior to testing, all HS-FRCM/HS-FRGM specimens underwent a 28-water curing. To assess freeze-thaw resistance, two commonly used parameters were introduced: compressive strength loss and mass loss. As demonstrated by Ebrahimi et al.^43^, the compressive strength loss and mass loss after freeze-thaw cycles can be calculated using Eqs. (1) and (2).

1 $$C1=\:\frac{C0-Cn}{C0}x100\%$$

2 $$M1=\:\frac{M0-Mn}{M0}x100\%$$

where; Cl​ represents the compressive strength loss (%), C0​ is the 28-day compressive strength (MPa), and Cn​ denotes the compressive strength (MPa) of the HS-FRCM/HS-FRGM specimens after n freeze-thaw cycles; Ml​ stands for the mass loss (%), M0​ is the 28-day mass (g), and Mn​ is the mass of the HS-FRCM/HS-FRGM specimens following n freeze-thaw cycles (g).

The HS-FRCM/HS-FRGM specimens (50 mm cubes) were also exposed to a high elevated temperature of up to 300 ^0^C for six hours, and the reduction in both compressive strength and mass was measured.

#### Microstructure characterization

Various microstructure characterizations were performed on HS-FRCM/HS-FRGM samples taken from specimens’ cubes which are cured in water. The analyses included SEM, mapping analysis, and FTIR. SEM imaging was performed after coating HS-FRCM/HS-FRGM samples after applying a 12 nm gold coating, using a JEOL JSM 6510 LV microscope operated at an acceleration voltage of 30 kV. Mapping analysis, performed with an Oxford X-Max 20 device, determined the atomic percentages of elements within the HS-FRCM/HS-FRGM matrix by different colors. FTIR spectra were recorded using the attenuated total reflection (ATR) technique across a wavenumber range of 400–4000 cm^−1^. FTIR spectroscopy was used to determine how the incorporation of VA and MW affected the hydration products by showing the functional groups of the HS-FRCM/HS-FRGM elements.

## Results and discussions

### Slump

**Fig. 6:**
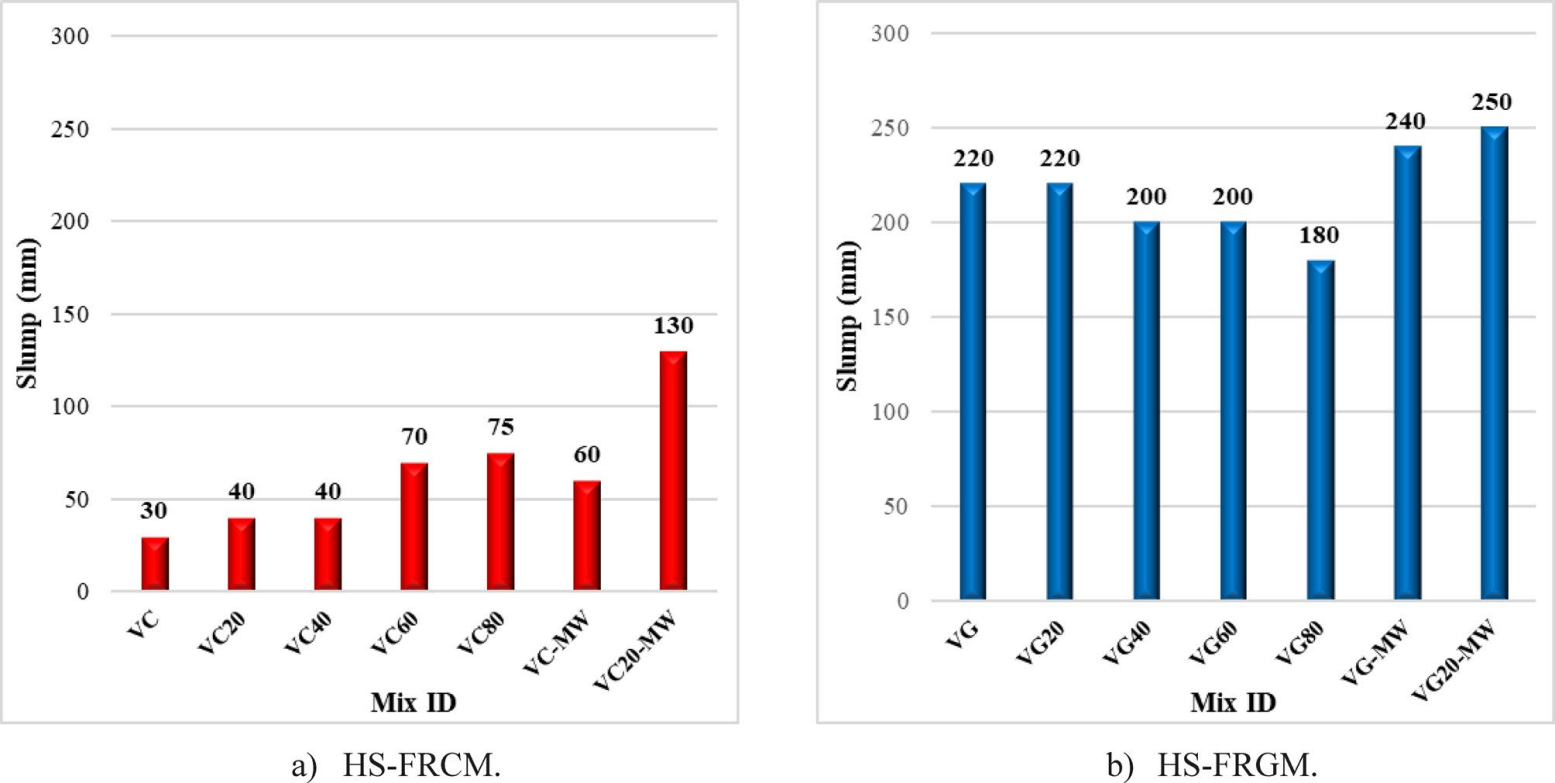
Slump values of HS-FRCM and HS-FRGM mixtures.

Table 4; Fig. 6a indicate the slump values of HS-FRCM mixtures. The results show that as the ratio of VA increased up to 80%, the slump value gradually enhanced. The slump values at 20%, 40%, 60%, and 80% VA increased by 33%, 33%, 133%, and 150%, respectively, compared to the mixture VC, which contains no VA. Additionally, the slump values increased when using MW in both mixtures VC and VC20. In mixture VC, the slump doubled when MW was used. For the mixture incorporating 20% VA and MW, a slump value of 130 mm was achieved, representing a significant positive effect compared to a similar mixture containing 20% VA but made with TW. The observed increase in slump values with the incorporation of VA may be attributed to the formation of spherical paste particles resulting from the blending of PC and VA. These spherical particles may act like tiny ball bearings within the mix, reducing internal friction and enhancing the flowability of the HS-FRCM mixtures containing VA. Another reason for the increased slump is the extremely fine particle size of fly ash, which facilitates the movement of water within the mix and consequently enhances the workability of HS-FRCM^44^. Additionally, the positive impact of using MW on the slump results in mixtures VC and VC20 can be explained by the disruption of water bonds. This process enables water to penetrate binder particles more effectively, accelerating the hydration process and subsequently increasing the slump compared to the use of TW^7,31^.

Figure 6b; Table 4 illustrate the slump values for HS-FRGM mixtures. It is evident that the presence of VA has a negative impact on slump when using up to 80% VA content, with decreases of 0%, 9%, 9%, and 18% observed at 20%, 40%, 60%, and 80% VA, respectively. However, in both mixtures VG and VG20, the use of MW demonstrated a positive effect, similar to that observed in corresponding HS-FRCM mixtures. The reduction in slump values observed with the use of VA is likely due to the rough and irregular texture of the VA particles, as illustrated in Fig. 2b. This texture can hinder water movement, leading to lower slump values. Conversely, the use of MW results in increased slump values because it enhances the preparation of the SH solution, which acted as an activator for geopolymeric reaction. This in turn improves hydration in HS-FRGM, resulting in higher slump values when MW was used^25,45^. It was evident that the slump values in HS-FRGM mixes were significantly higher than those in HS-FRCM mixes across all tested ratios of VA, with HS-FRGM slump being up to seven times greater than those of HS-FRCM. This indicates that the components of HS-FRGM mixes positively influence its workability. This improvement may be attributed to the effect of the AA solution, which enhances the matrix’s viscosity and internal lubrication, thereby increasing the workability of HS-FRGM^45^.

**Table 4:** Percentage-based relative values of the fresh and hardened characteristics for all HS-FRCM and HS-FRGM mixtures.

**Group name**	**Mix ID**	**Slump** **(mm)**	**Compressive strength (%)**						**Flexural ** **strength (%)**	**Tensile ** **strength (%)**
			**7 days**	**28 days**				**90 days**	**28 days**	**28 days**
				**Tap water**	**Air**	**Sunlight**	**Seawater**			
**HS-FRCM**	**VC**	100	100	100	100	100	100	100	100	100
	**VC20**	133	91	100	109	90	93	109	86	85
	**VC40**	133	69	70	--	--	--	89	86	80
	**VC60**	233	35	45	--	--	--	58	71	70
	**VC80**	250	15	22	--	--	--	32	71	67
	**VC-MW**	200	102	95	109	106	96	97	157	105
	**VC20-MW**	433	107	106	117	98	93	114	114	97
**HS-FRGM**	**VG**	100	100	100	100	100	100	100	100	100
	**VG20**	100	93	94	95	86	132	99	83	94
	**VG40**	91	78	86	--	--	--	96	83	89
	**VG60**	91	42	69	--	--	--	79	75	86
	**VG80**	82	30	46	--	--	--	46	67	62
	**VG-MW**	109	115	106	106	110	147	107	100	113
	**VG20-MW**	114	95	97	103	92	106	104	92	109

### Hardened characteristics

#### Compressive strength results

**Fig. 7:**
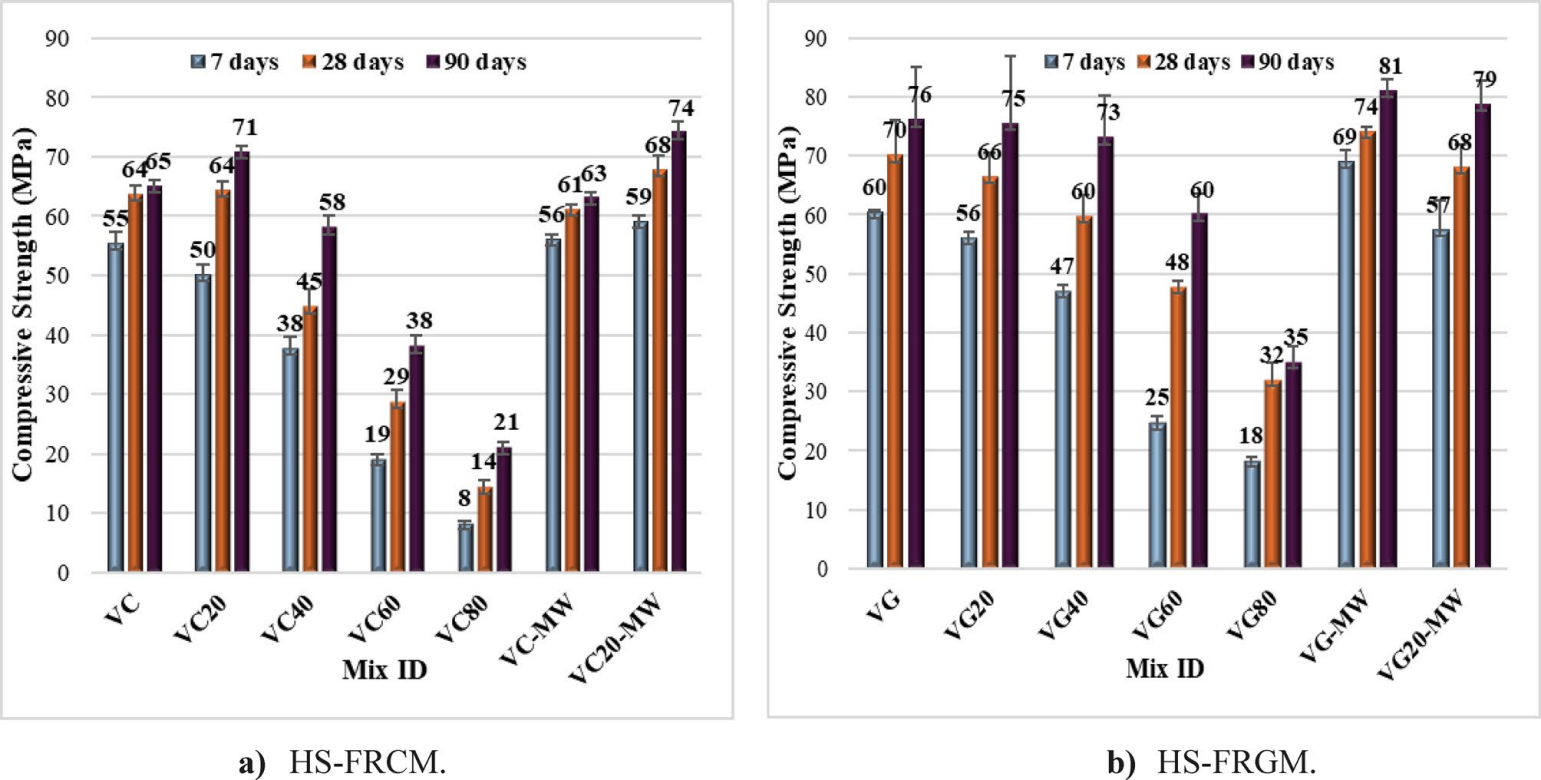
Impact of VA and MW on HS-FRCM/HS-FRGM compressive strength (tap water curing).

Figure 7; Table 4 show the HS-FRCM and HS-FRGM compressive strength variation at different VA ratios measured at 7, 28, and 90 days of conventional tap water curing. As shown in Fig. 7a, for the HS-FRCM mixtures at 7 days, the compressive strength decreased by 9%, 31%, 65%, and 85% for 20%, 40%, 60%, and 80% VA, respectively, in comparison with the control HS-FRCM mix. At 28 days, the compressive strength decreased by 0%, 30%, 55%, and 78% for the same VA ratios, respectively. At 90 days, the compressive strength increased by 9% for the 20% VA, while it decreased by 11%, 42%, and 68% for the 40%, 60%, and 80% VA, respectively. The reduction in compressive strength at different levels of VA may be due to the reduction in PC and GGBFS contents which might cause ineffective hydration process^8,46,47^. Figure 7a shows also the impact of using MW on HS-FRCM compressive strength (see mixes VC-MW and VC20-MW). As shown, MW had no impact on HS-FRCM compressive strength at 7, 28, or 90 days for the control mix. However, in the mix containing 20% VA, the use of MW had a positive effect, increasing compressive strength by 18%, 6%, and 4% at 7, 28, and 90 days, respectively. This positive effect of MW can be attributed to the ability of magnetic field to break the bonds between water molecules, allowing the water to penetrate the fine material particles more easily. Consequently, the effectiveness of the hydration process was enhanced, resulting in increased compressive strength^32,48^. This phenomenon occurred also in the mixture with 20% VA due to the pozzolanic reaction of VA with the calcium hydroxide (CH) produced during the hydration phase between water and PC, GGBFS, and VA, leading to the formation of calcium silicate hydrates (C-S-H). These hydrates were further crystallized by the magnetic field to fill the voids at the microscopic level, thereby significantly enhanced the HS-FRCM compressive strength^31,49^.

In HS-FRGM mixtures shown in Fig. 7b, the same trend was observed. As the percentage of VA increased, the compressive strength decreased, particularly at early ages. At 7 days, compressive strength decreased by 7%, 22%, 58%, and 70% for 20%, 40%, 60%, and 80% VA, respectively. At 28 days, compressive strength decreased by 6%, 14%, 31%, and 54% for the same ratios of VA, respectively. However, at 90 days, the 20% and 40% VA showed approximately no effect in compressive strength compared to the control mixture. These results indicate that as the curing age increases, the negative impact of increased VA content diminishes. This could be attributed to the long-term pozzolanic effects of VA due to its high pozzolanic elements of SiO_2_ and Al_2_O_3_ content^9,50^. MW also had a positive effect on mixtures VG and VG20 of the HS-FRGM mixes, as illustrated in Fig. 7b. At 7, 28, and 90 days for the VG mixture, compressive strength improved by 15%, 6%, and 7%, respectively. In contrast, for VG20 mixture, compressive strength improved by 5% at 90 days, while there was no effect at 7 and 28 days. The positive impact of MW on increasing compressive strength can be attributed to the effectiveness of the magnetization of water used to prepare the AA^25^.

In general, HS-FRGM mixtures exhibited higher compressive strengths than HS-FRCM mixtures, particularly at high levels of VA and at later curing ages. This can be attributed to the pozzolanic reaction between the VA and AA, which resulted in the formation of a dense paste with reduced voids, thereby enhanced compressive strength^51^. This also demonstrates a positive effect on compressive strength at various ages in the VC20, VG, and VG20 mixtures. The observed improvement can be attributed to several key factors. First, the VC20 mix contains VA, which replaces 20% of the PC and GGBFS. As shown in Table 1, the SiO₂ content in VA is nearly double that found in GGBFS. This higher silica content enhances the hydration process when GGBFS is partially replaced with VA, especially in the presence of MW. The reaction between VA and the free CH generated during hydration produces additional gel-like compounds, which are further promoted by MW due to its superior ability to break molecular bonds and deeply penetrate fine materials. A similar mechanism applies to the VG and VG20 mixtures, which contain FA, GGBFS, and VA. These mixtures have sufficient SiO₂ content to activate and enhance the hydration process in the presence of MW, resulting in improved mechanical performance.

**Fig. 8:**
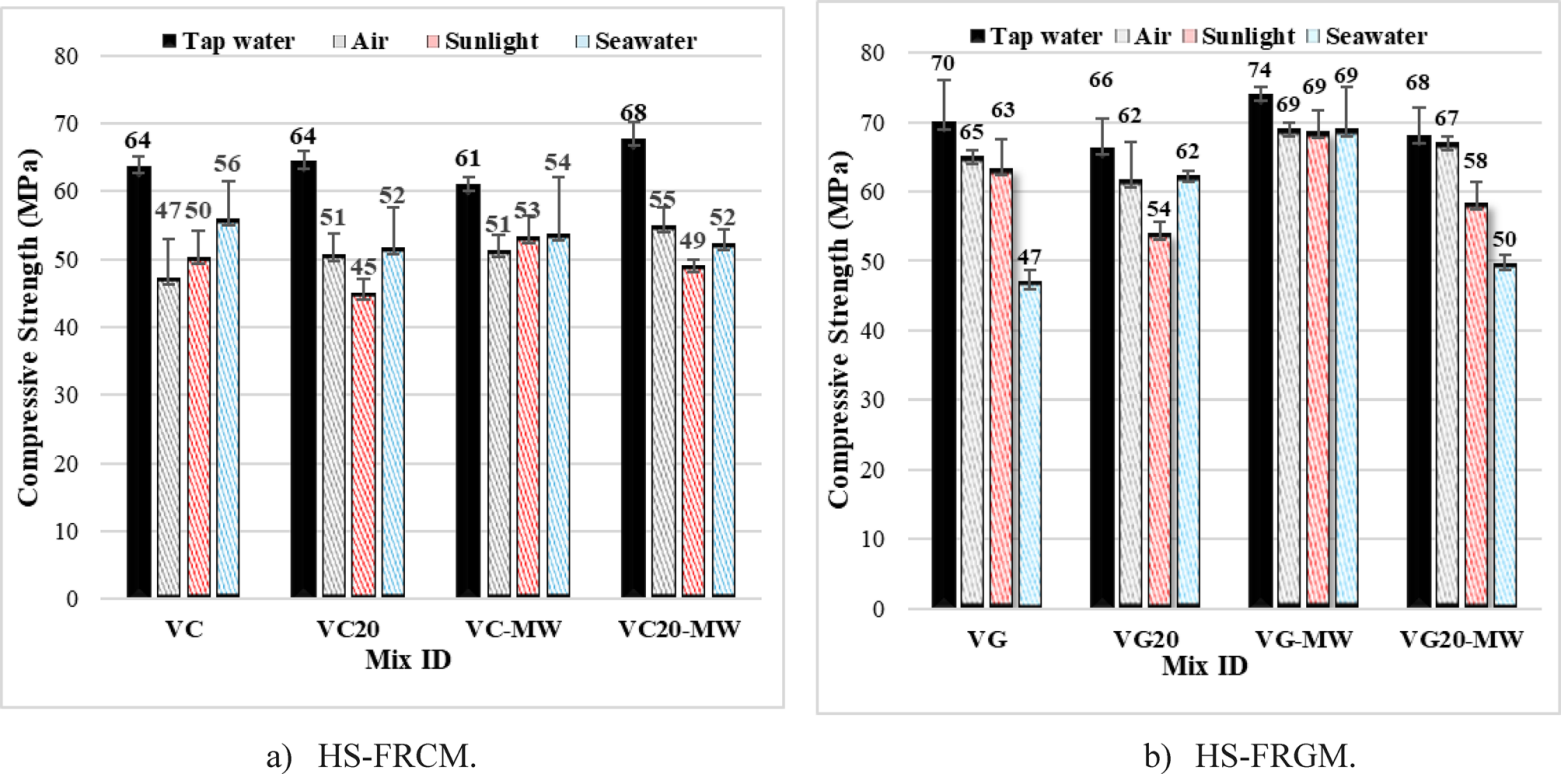
Effect of curing method on HS-FRCM/HS-FRGM compressive strength (at 28 days).

Figure 8; Table 4 illustrate the impact of curing conditions on the compressive strength of HS-FRCM/HS-FRGM at 28 days. The different curing conditions (tap water, air, sunlight, and seawater) were applied on the control mixes and those incorporating 20% VA. This was measured for those mixes made with TW as well as MW. As shown in Fig. 8a, it is evident that in the HS-FRCM control mix that the compressive strength decreased when cured in air, sunlight, and seawater by 27%, 22%, and 13%, respectively, in comparison with curing in tap water. For the mix with 20% VA, the compressive strength decreased by 20%, 30%, and 19%, respectively, compared to curing in tap water. In mixes made with MW, the compressive strength decreased by 16%, 13%, and 11%, respectively for the control mix, and by 19%, 28%, and 24%, respectively for the 20% VA mix under air, sunlight, and seawater curing conditions. These results indicate the effectiveness of using tap water in curing HS-FRCM compared to any other curing method. This may be due to the complete hydration occurrence during using tap water for PC, GGBFS, and VA. It worth noting that using any curing method proposed in this study could keep the value of HS-FRCM compressive strength higher than 45 MPa regardless the existence of VA or MW.

Figure 8b illustrates the reduction in HS-FRGM compressive strength resulting from the use of various curing methods compared to traditional tap water curing for both the HS-FRCM control mix and the HS-FRGM mix containing 20% VA. It is evident from the figure that in the HS-FRGM control mix, the compressive strength decreased by 7%, 10%, and 33% when cured in air, sunlight, and seawater, respectively. In the HS-FRGM mix containing 20% VA, the compressive strength decreased by 6%, 18%, and 6% under the same curing conditions, respectively. For the control HS-FRGM made with MW, the compressive strength decreased by only 7% across the three different curing environments compared to traditional tap water curing. Additionally, in the HS-FRGM mix containing 20% VA, the compressive strength decreased by 1%, 15%, and 26% when cured in air, sunlight, and seawater, respectively. It can be concluded that all applied curing conditions could positively affect the HS-FRGM compressive strength, especially using seawater curing. This is due to the densification of the microstructure and improved integrity of the hydrated geopolymer materials. The obvious HS-FRGM compressive strength enhancement when cured in seawater may be because of the formation of Friedel’s salt, which occurs when VA interacts with seawater during the process of curing, leading to a denser paste and fewer pores, and consequently compressive strength increase^51^.

#### Flexural strength results

**Fig. 9:**
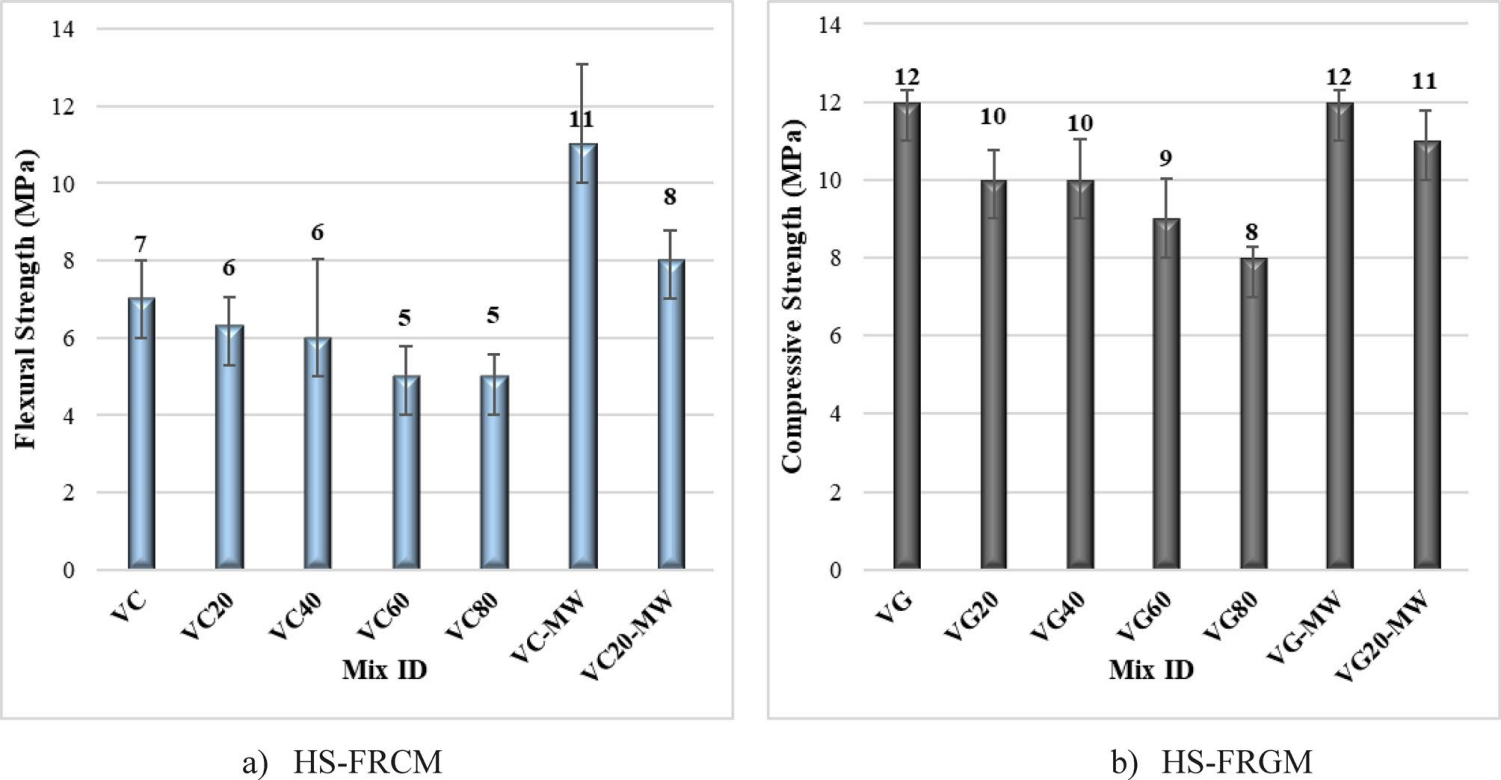
Impact of VA and MW on HS-FRCM/HS-FRGM flexural strength (tap water curing).

Figure 9; Table 4 illustrate the flexural strength of both HS-FRCM and HS-FRGM mixtures at VA contents of 0%, 20%, 40%, 60%, and 80%, as well as the impact of MW on mixtures containing 0% and 20% VA. It is evident from Fig. 9a that the flexural strength decreased by 14%, 14%, 29%, and 29% at 20%, 40%, 60%, and 80% VA ratios, respectively, for the HS-FRCM mixtures compared to control mix. The reduction in flexural strength with the increased VA content may be because of the decreased PC and GGBFS, leading to a diminished effectiveness of the hydration process. In contrast, the impact of MW on the mixes VC and VC20 was highly positive, resulting in increases in flexural strength of 57% and 33%, respectively. The positive impact of MW on flexural strength can be explained by the magnetic field and its ability to disrupt the bonds between water molecules. This facilitates better penetration between the binder materials, thereby enhancing the efficiency of the hydration process and increasing the flexural strength^52,53^. The same trend was also observed in HS-FRGM mixtures shown in Fig. 9b, as the flexural strength decreased at 20%, 40%, 60%, 80% VA by 17%, 17%, 25%, and 33%, respectively. This is due to the low efficiency of hydration process using AA in HS-FRGM mixing because of the lower content of FA and GGBFS. The MW had no effect on the flexural strength of VG or VG20 mixes. Also, all HS-FRCM/HS-FRGM mixtures have a value of flexural strength above 5 MPa due to the existence of GF which in turn, controls the width and propagation of cracks, thereby providing a high value for flexural strength^54^.

#### Uniaxial tensile strength results

**Fig. 10:**
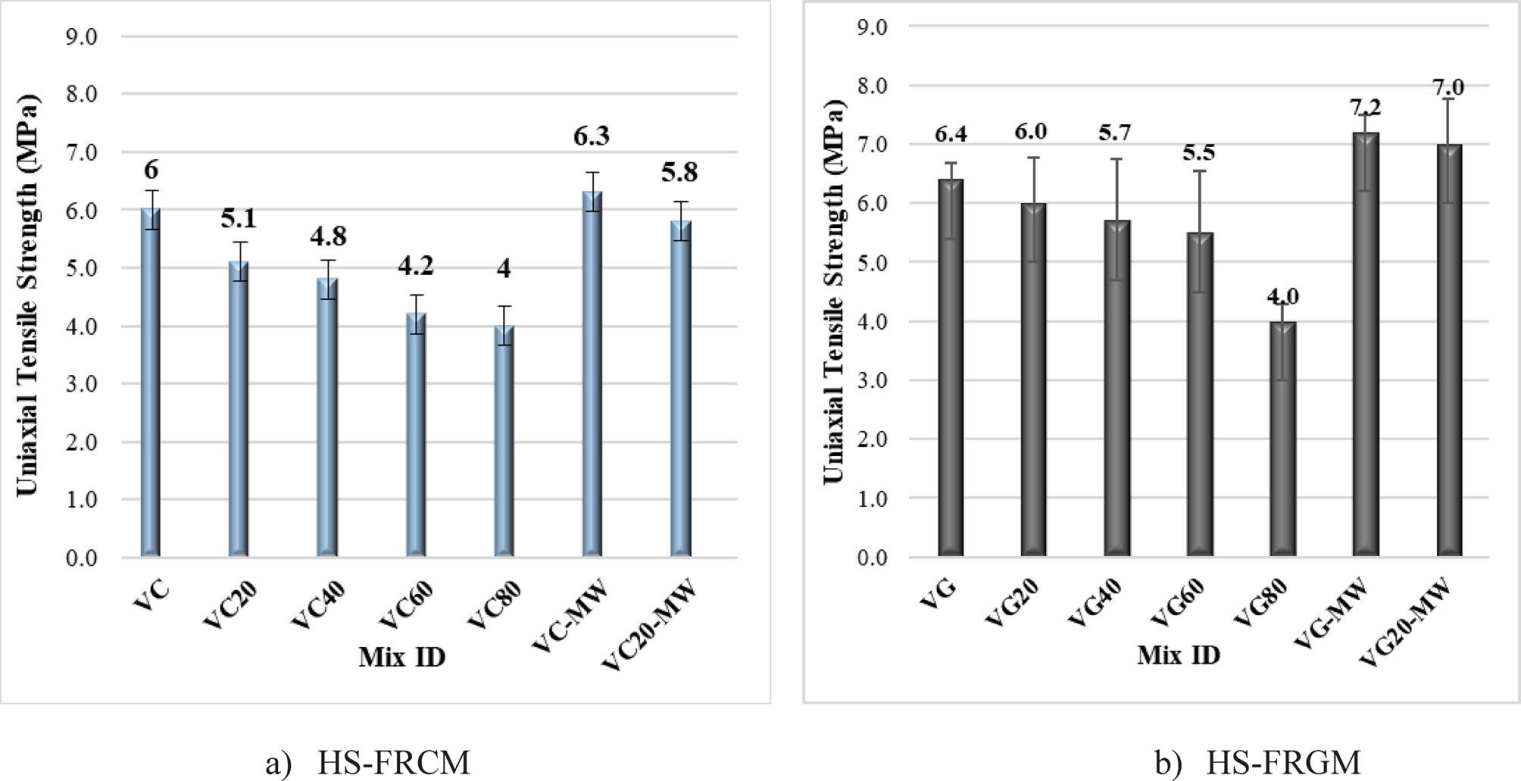
Effect of VA and MW on HS-FRCM/HS-FRGM uniaxial tensile strength (tap water curing).

Figure 10; Table 4 illustrate the uniaxial tensile strength values for both HS-FRCM and HS-FRGM mixtures at VA contents of 0%, 20%, 40%, 60%, and 80%, as well as the impact of MW on the mixes containing 0% and 20% VA. It is evident from Fig. 10a that the HS-FRCM uniaxial tensile strength decreased by 15%, 20%, 30%, and 33% at the 20%, 40%, 60%, and 80% VA ratios, respectively. This reduction may be because the fact that as the PC and GGBFS content decreases, the efficiency of the hydration process diminishes. Additionally, it is noted that the effect of MW is negligible on both HS-FRCM control mix and the 20% VA mix. This is likely due to the small thickness of the samples used in the uniaxial tensile strength test (13 mm) compared to those used in compression tests, which results in fracture occurring at values that are quite similar. For HS-FRGM mixtures, it is noticed in Fig. 10b that the uniaxial tensile strength decreased by 6%, 11%, 14%, and 38% at 20%, 40%, 60%, and 80% VA, respectively. In mixtures VG and VG20, the MW slightly enhanced the uniaxial tensile strength by up to 17% compared to using TW. The enhancement in uniaxial tensile strength of HS-FRGM mixtures may be due to the relatively denser geopolymer paste made with MW-based AA. Also, all HS-FRCM/HS-FRGM mixtures have a value of uniaxial tensile above 4 MPa due to the existence of GF which in turn, controls the width and propagation of cracks, thereby providing a high value for uniaxial tensile^54^.

### Durability evaluation

#### Effect of high elevated temperature

**Fig. 11:**
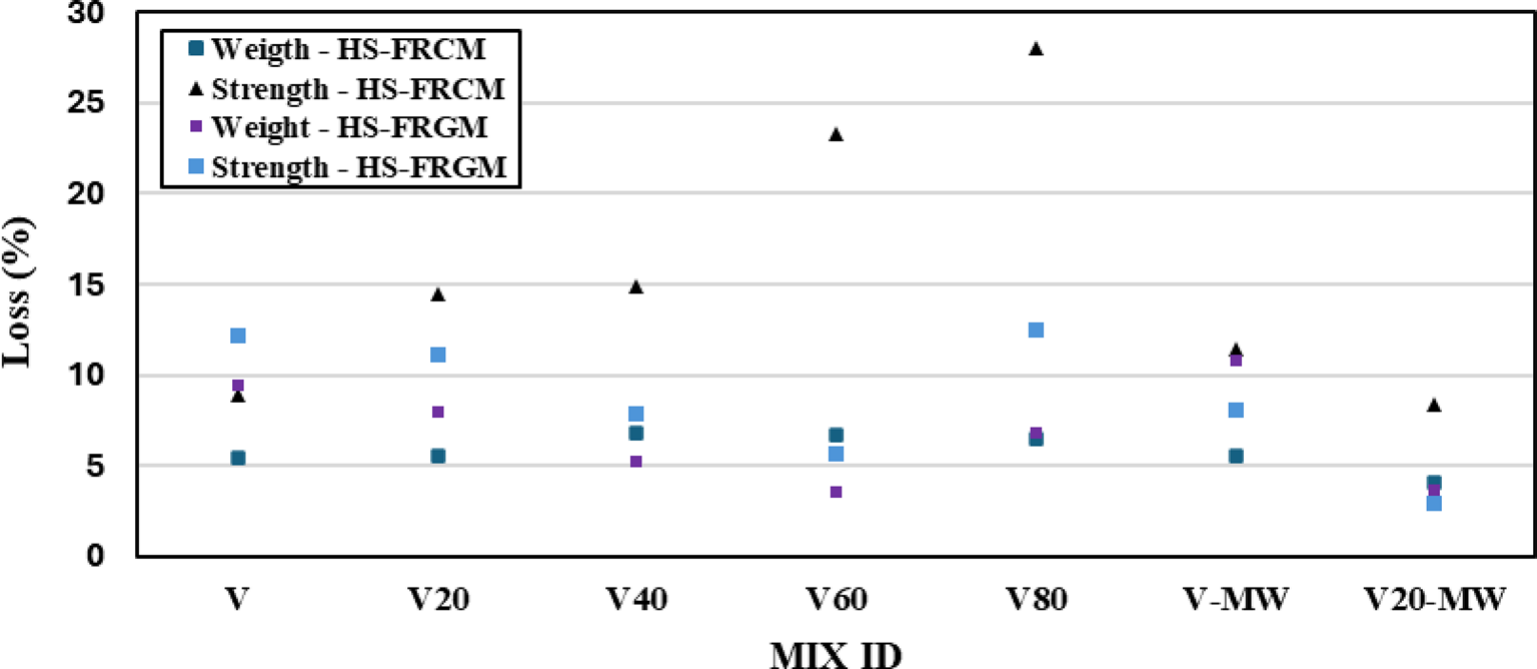
Effect of elevated temperature on the weight and compressive strength of HS-FRCM/HS-FRGM.

In Fig. 11, the effect of elevated temperature at 300 °C on weight loss and compressive strength loss of HS-FRCM and HS-FRGM mixtures (at 28 days of water curing) is shown. For the HS-FRCM mixtures, the weight loss values at VA contents of 0%, 20%, 40%, 60%, and 80% were 5%, 6%, 7%, 7%, and 6%, respectively. These values indicate a relatively stable weight loss for the HS-FRCM mixtures. For HS-FRCM mixtures made with MW (VC and VC20), it is observed that the mix VC remained unaffected by the temperature up to 300 °C. However, mix VC20 showed a reduction in weight loss from 6 to 4% (33% decrease). This demonstrates the effectiveness of MW in preserving the HS-FRCM structure while exposure to high temperatures. It is also noted that when HS-FRCM exposed to high temperatures for 6 h, the loss of strength for mixtures containing 20%, 40%, 60%, and 80% VA increased by 15%, 15%, 23%, and 28%, respectively, compared to the losses shown by the control HS-FRCM mixture (9%). This is due to the slower rate of hydration and chemical bonding that occurs because of the rapid cooling provided by the water, along with the abrupt temperature differential between the surface and core of the specimen caused by water cooling, leads to the formation of microcracks^55^. Meanwhile, a positive effect of MW is observed in mixture VC20, where the loss in compressive strength decreases from 15 to 8% when using MW compared to the use of TW.

On the other hand, it is evident from Fig. 11 that the results of HS-FRGM mixtures under elevated temperature are more stable compared to the corresponding HS-FRCM mixtures, particularly the weight loss. The weight loss decreased by 11%, 44%, 55%, and 23% at VA contents of 20%, 40%, 60%, and 80%, respectively. This indicates that VA has a positive effect on weight loss of HS-FRGM blends after high-temperature exposure. This can be attributed to three reasons. The first reason is that the geopolymer paste is denser than the cement paste, making it more difficult to break down its hydration components, as they are strengthened from the outset by the high AA influencing the hydration process^25^. The second reason is that VA contains pozzolanic elements which participate in the hydration process in the presence of the AA. The third reason is the nature of VA in which it is sourced from volcanic eruption at very high temperatures. The positive effect of MW is evident in mixture VG20 as when MW was used, the weight loss was only 4% compared with 8% weight loss when TW was used. This is attributed to the fact that MW stabilizes the geopolymer paste, as it enhances the strength of the AA compared to the TW-based AA. Similarly, the loss of HS-FRGM compressive strength decreased for the mixtures containing 20%, 40%, and 60% VA by 8%, 33%, and 50%, respectively. In contrast, the loss of compressive strength increased by 8% for the mixture with 80% VA. The positive impact of MW is quite evident in HS-FRGM mixtures containing 0% and 20% VA, where the loss in compressive strength decreased by 33% and 73%, respectively. This can be attributed to the effectiveness of MW in enhancing the AA reactivity during the hydration process of the geopolymer paste.

#### Effect of rapid freeze-thaw cycles

**Fig. 12:**
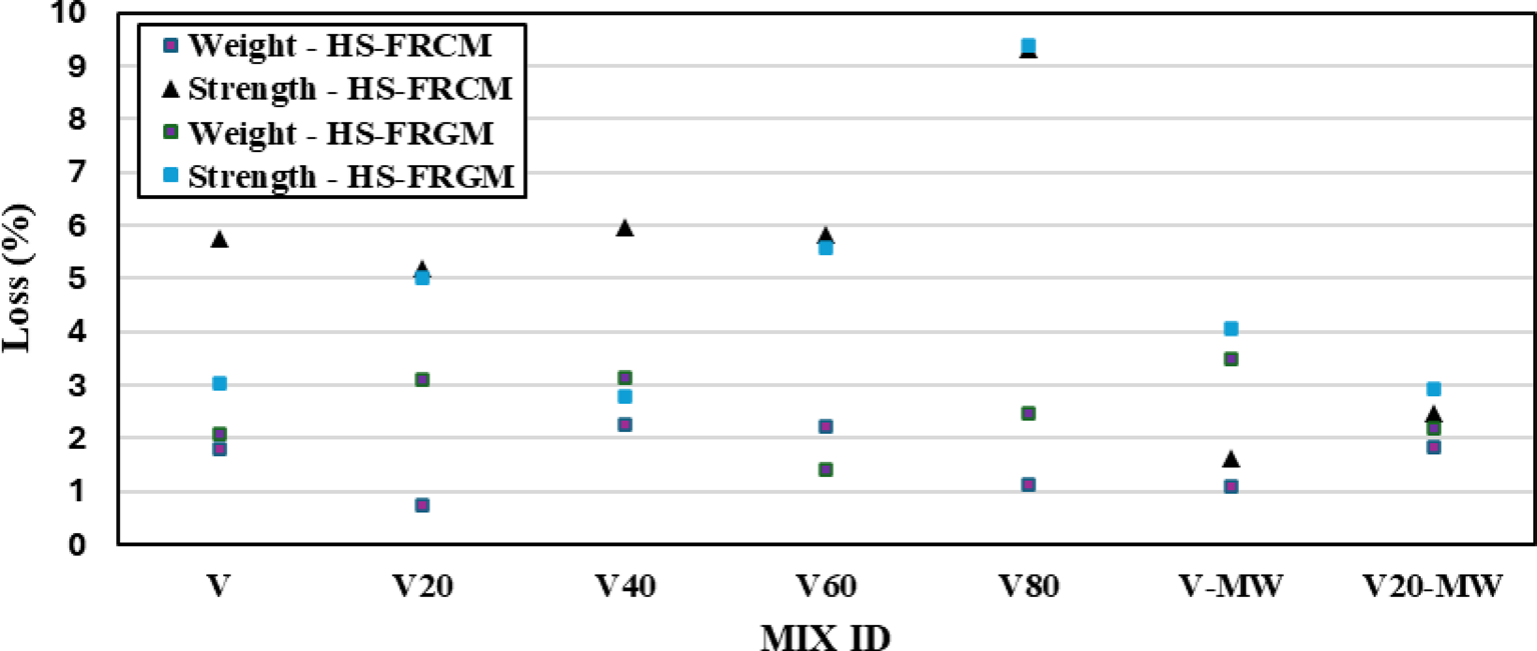
Effect of rapid freezing and thawing cycles on the weight and compressive strength of HS-FRCM/HS-FRGM.

Figure 12 illustrates the weight loss and compressive strength loss in HS-FRCM and HS-FRGM mixes after being subjected to 300 rapid freeze-thaw cycles (after 28 days of tap water curing). Initially, for the HS-FRCM mixes, regardless of the presence of VA or MW, the weight loss ranged between 1% and 2%, which is relatively low considering the impact of 300 freeze-thaw cycles. It can be concluded that VA has a positive and significant effect on freeze-thaw resistance. It is also observed that the strength losses in HS-FRCM mixes containing 0%, 20%, 40%, 60%, and 80% VA were 6%, 5%, 6%, 6%, and 9%, respectively, as a result of the freeze-thaw cycles. This means that compared to the control mix, HS-FRCM mixes containing 20–60% VA were less affected by freeze-thaw cycles due to the formation of new C–S–H gels in the matrix, through a pozzolanic reaction with portlandite (a product of cement hydration). This allowed the matrix to develop additional strength and enhanced their compressive capacity after freeze-thaw cycles^56^. However, at 80% VA content, the compressive strength decreased by 9%, which is due to the low C-S-H gels by the reduction of PC content. The positive effect of MW is also evident in mixes VC and VC20, where the compressive strength loss after freeze-thaw cycles was only 2%, compared with the corresponding mixes made with TW (showed 7% strength losses). This demonstrates the beneficial effect of using both VA and MW in the VC20 mix in this study, which was almost unaffected by the freeze-thaw cycles. On the other hand, for the HS-FRGM mixes, the weight loss in all mixes ranged from 2 to 3%, regardless the existence of VA or MW, which is similar to the behavior of the corresponding HS-FRCM mixes. Similarly, the compressive strength losses in HS-FRGM mixes containing 0%, 20%, 40%, 60%, and 80% VA were 3%, 5%, 3%, 6%, and 9%, respectively, which followed the same trend observed in the HS-FRCM mixes.

### Microstructure characterizations

#### Scanning electronic microscope (SEM) analysis

**Fig. 13:**
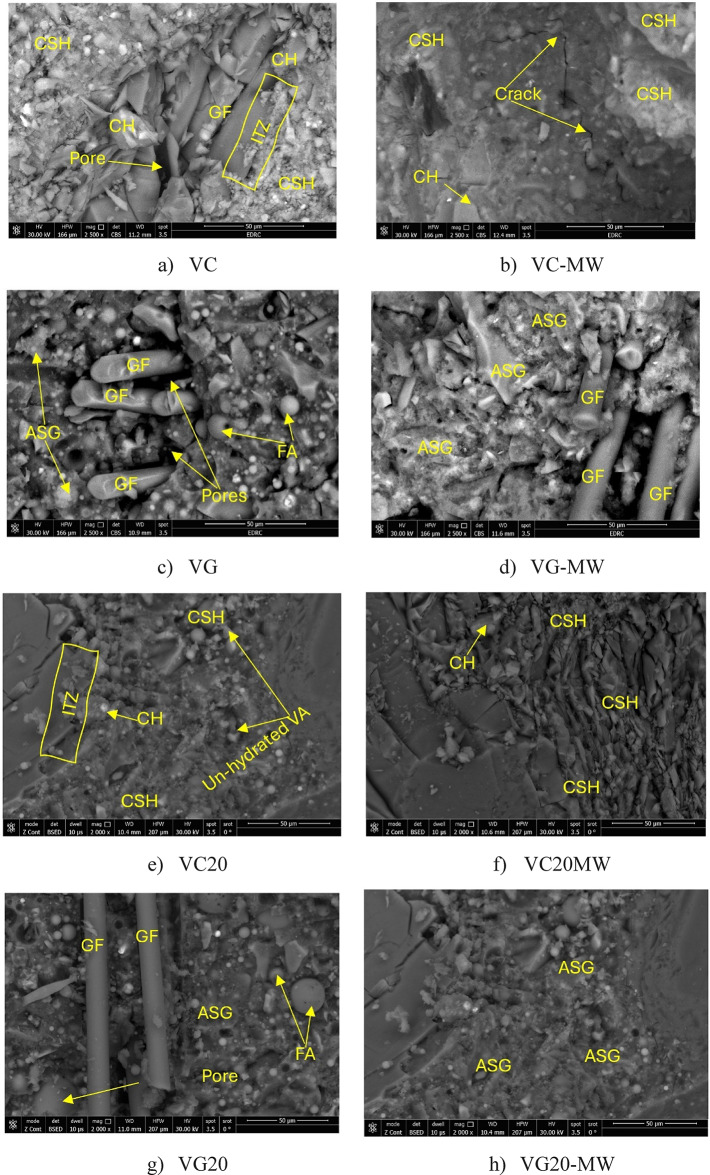
SEM analysis of HS-FRCM/HS-FRGM mixtures.

The SEM analysis in this study is conducted to examine the effects of MW and VA on the characteristics of the cementitious and geopolymer paste within HS-FRCM and HS-FRGM mixtures. Figure 13 presents the surface morphology of HS-FRCM and HS-FRGM mixtures containing 0% and 20% VA, utilizing TW or MW. In general, the primary binder phase in HS-FRCM mixtures is C-S-H-, while in HS-FRGM mixtures, due to the minimal calcium content, the binding phase is aluminosilicate gel (ASG). A comparison of the SEM images in Fig. 13 reveals that the geopolymeric paste in HS-FRGM mixtures exhibits greater cohesion and lower porosity than the cementitious paste in HS-FRCM mixtures, which correlates with the superior mechanical properties observed in HS-FRGM mixes. Furthermore, the GF are more firmly dispersed in the geopolymeric matrix of HS-FRGM mixes, contributing to their higher flexural strength. The presence of spherical particles, likely representing FA, in HS-FRGM mixes further explains the higher slump values of these mixes, as these particles facilitate easier movability of water over them.

In mixtures prepared with MW, the SEM analysis shows an increased presence of CSH in the HS-FRCM mixes and ASG in the HS-FRGM mixes. This may be because of the enhanced hydration phase promoted by MW, which accelerates the formation of hydration products that improve cohesion. Furthermore, in mixes prepared with TW + VA, calcium hydroxide crystals are more prominent at the interfacial transition zone (ITZ) between the GF and HS-FRCM, compared to the regions further from the ITZ. However, in the MW + VA mixes, the ITZ is less distinct, suggesting that the MW treatment facilitates more effective reactions between CH and the cementitious materials, promoting the formation of C-S-H. These results indicate that geopolymeric materials typically show improved paste morphology and mechanical properties, thanks to their strong cohesive nature, which is enhanced by the presence of the AA that stimulates the geopolymerization reaction, as well as the positive impact of MW.

The analysis also reveals a correlation between the mechanical and durability properties of the HS-FRCM/HS-FRGM mixes with varying VA contents (0% and 20%) and MW, as well as the fracture surface morphology. For instance, the VC20-MW mixture shows an improvement in compressive and flexural strengths at 28 days (the same curing age as the SEM analysis), when compared to the VC20 mixture. These improvements are consistent with the microstructural observations, which show a reduced number of voids and a greater presence of C-S-H gel, as illustrated in Fig. 13. Similar trends are observed for the VG20 and VG20-MW mixtures, with compressive strength increases after the employment of MW, due to the enhanced geopolymeric reaction of ASG in the VG20-MW mixture compared to the VG20 mixture.

#### Mapping analysis

**Fig. 14:**
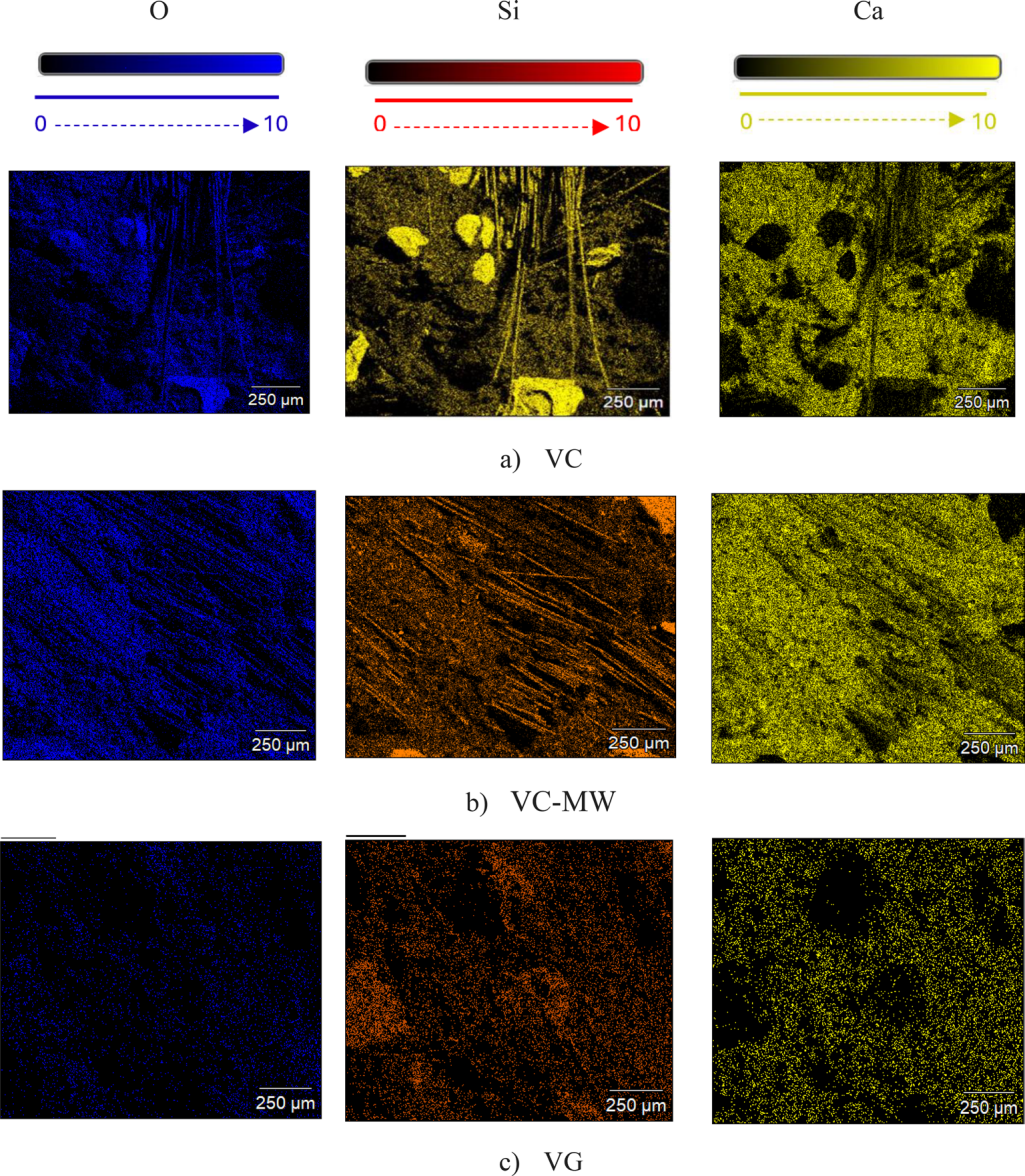

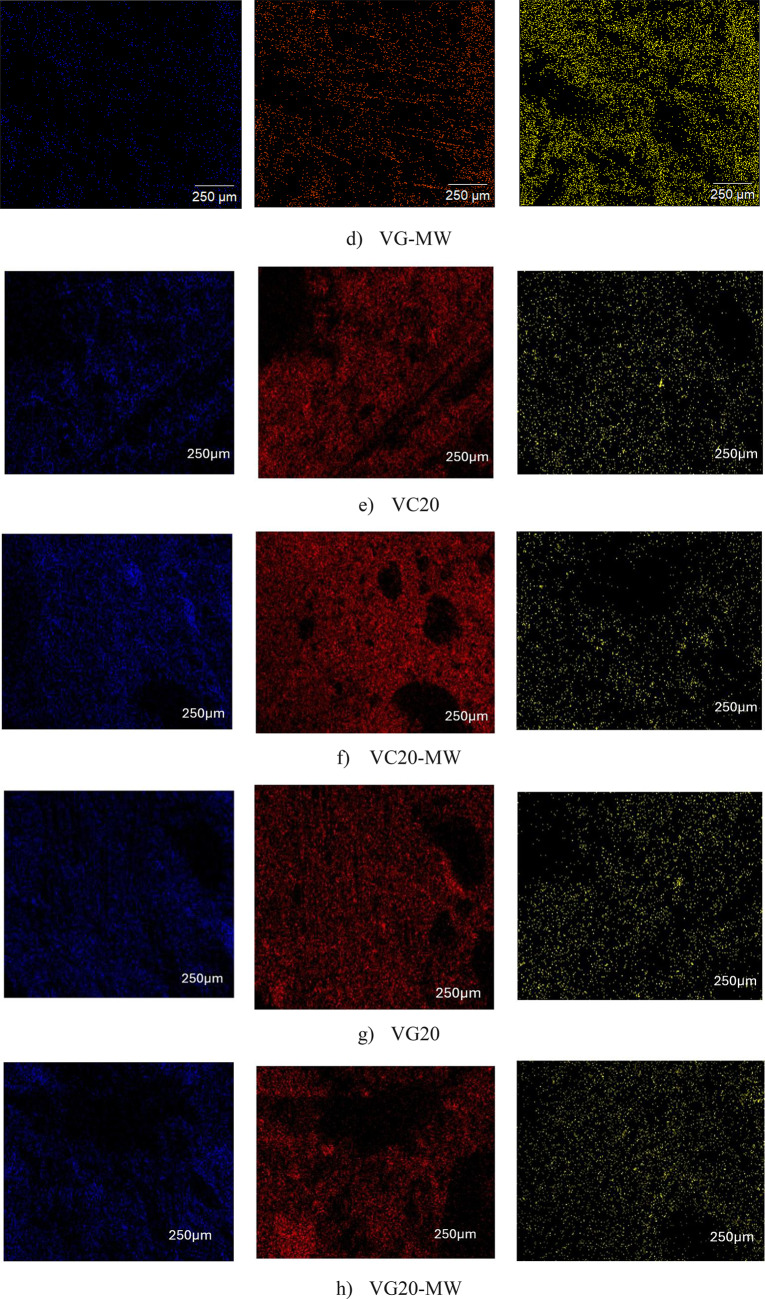
Mapping analysis of HS-FRCM/HS-FRGM mixtures.

Table 5; Fig. 14 illustrate the distribution of chemical elements in HS-FRCM and HS-FRGM mixtures with 0% and 20% VA content, with and without using MW. Table 5 presents the percentages of chemical elements in the mixtures, including oxygen, silica, and calcium, as well as the ratio of calcium to silica (Ca/Si). This ratio is of particular interest because it is related to the mechanical and durability properties of concrete due to the higher content of Si during the hydration process, the higher presence of CSH in HS-FRCM or ASG in HS-FRGM, and both are responsible of binding of cement paste or geopolymer paste. Figure 14 further demonstrates the distribution of these elements by showing the extension of each element in each mixture. Therefore, it can be concluded that there is an inverse relationship between the Ca/Si and the mechanical properties of the HS-FRCM and HS-FRGM mixtures. For example, the Ca/Si in the HS-FRCM control mixture is 3.88, and this ratio decreased to 1.47 in mixture VC20-MW. This reduction can be linked to the improved mechanical properties of VC20-MW mixture, which is the highest performing HS-FRCM mixture in terms of these properties. Similarly, the Ca/Si decreased from 0.78 in the HS-FRGM control mixture to 0.48 in VG20-MW mixture, which shows the best overall mechanical and durability properties in this study.

In general, the Ca/Si ratio in HS-FRCM mixtures is observed to be relatively higher than in HS-FRGM mixtures. This is primarily due to the presence of PC in HS-FRCM, which has a comparatively high CaO content. However, in the VC20 and VG20 mixtures, a reduction in the Ca/Si ratio is noted when MW is used. This correlates well with the observed increase in compressive strength and overall mechanical performance in these mixtures compared to those using TW. This improvement can be explained by the enhanced hydration process facilitated by MW, which activates unreacted VA particles that may remain inert under conventional curing with TW. As a result, the reduced Ca/Si ratio reflects a more complete pozzolanic reaction, leading to better mechanical performance. Thus, there is an inverse relationship between the Ca/Si ratio and strength development. The observed improvement underscores the role of MW in fully activating the pozzolanic potential of VA in both HS-FRCM and HS-FRGM systems. It is also evident from Fig. 14a that calcium dominates the control mix in the HS-FRCM mixtures, unlike the other mixes. This confirms the effectiveness of both VA and MW in enhancing the presence of silica during the hydration process in other mixes, thereby improving the properties. Therefore, it can be concluded that there is a strong relationship between the macroscale and microscale properties of HS-FRCM and HS-FRGM, which supports the validity of the experimental findings of this study.

**Table 5:** Chemical elements of HS-FRCM/HS-FRGM using mapping analysis.

Mix ID	Elements			
	O	Si	Ca	Ca/Si
VC	40.3	10.2	39.6	3.88
VC-MW	44.5	10.7	31.5	2.94
VC20	41.8	7.16	37.67	5.26
VC20-MW	48.66	13.89	20.5	1.47
VG	45.2	19.8	15.6	0.78
VG-MW	44.8	10.8	6.1	0.56
VG20	42.51	27.72	16.53	0.59
VG20-MW	49.36	22.63	10.91	0.48

#### Fourier-transform infrared (FTIR) spectroscopy analysis

**Fig. 15:**
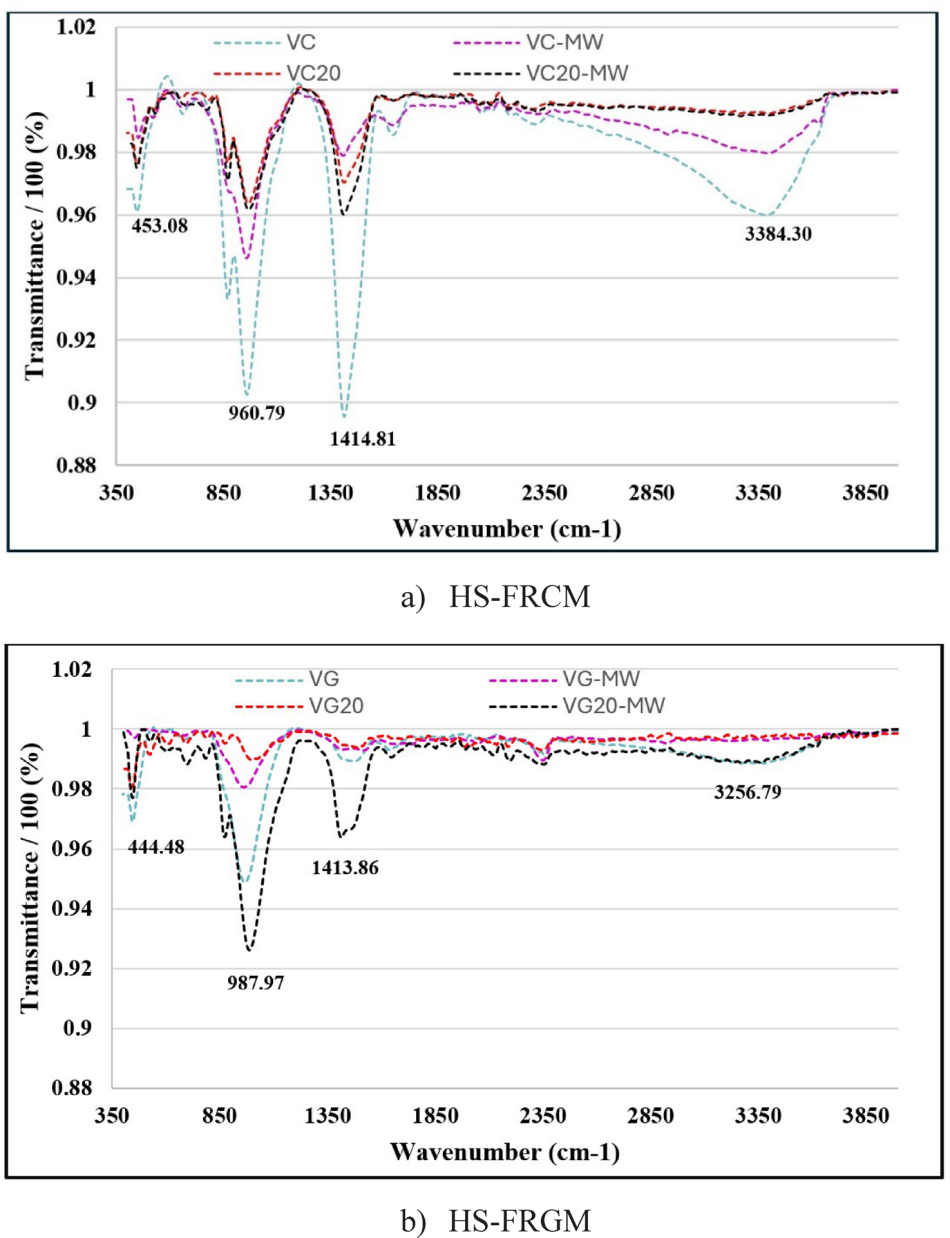
FTIR of HS-FRCM/HS-FRGM mixtures.

FTIR analysis was used to identify specific functional groups in the HS-FRCM/HS-FRGM mixtures. Figure 15a presents the FTIR transmission spectra of the 28-day hydrated samples, including VC, VC-MW, VC20, VC20-MW, VG, VG-MW, VG20, VG20-MW. Each FTIR spectrum exhibited prominent absorption bands at 453, 960, 1414, 3385 cm^−1^ for HS-FRCM mixtures, and 445, 987, 1414, 3256 cm^−1^ for HS-FRGM mixtures. The wavenumbers around 450 cm^−1^ and 960 cm^−1^ correspond to the characteristic absorption peaks associated with Si-O bending vibrations and Si-O symmetric stretching vibrations, respectively. The wavenumber associated with the Si-O symmetric stretching vibration near 960 cm^−1^ increased as the replacement of PC + GGBFS with VA increased. The peak around 3400 cm^−1^ corresponds to the O-H vibration in Ca(OH)₂. This peak decreased as the content of VA increased, indicating a reduction in the amount of CH in the mixture. The reduction in the CH peaks is attributed to two factors: the dilution effect of PC + GGBFS due to the addition of VA and the pozzolanic activity of VA. In the latter, SiO_2_ from VA reacts with CH to form C-S-H gel, thereby consuming some of the CH and further contributing to the decrease in its peaks^57^. The shift and intensity of the bands reflect the dual impact of both VA content and the use of MW, which appear to improve the reaction kinetics and structure of the cementitious and geopolymer matrix.

Figure 15b presents the FTIR spectra for four different HS-FRGM mixes: VG, VG20, VG-MW, and VG20-MW. The FTIR spectra display several characteristic absorption peaks at 444.48 cm⁻¹, 987.97 cm⁻¹, 1413.86 cm⁻¹, and 3256.79 cm⁻¹. The band around 444 cm⁻¹ is typically attributed to Si–O bending vibrations, while the peak near 987 cm⁻¹ corresponds to asymmetric stretching vibrations of Si–O–T (T = Si or Al), indicating the extent of geopolymeric gel formation. The absorption at 1413.86 cm⁻¹ may be associated with C–O stretching, possibly due to carbonate presence or atmospheric CO₂ interactions. The broad band at 3256.79 cm⁻¹ is related to O–H stretching vibrations, suggesting the presence of hydroxyl groups or bound water. Among the mixes, the VG20-MW sample exhibited more intense and sharper peaks, especially at 987 and 1413 cm⁻¹, indicating an enhanced degree of geopolymerization and behavior development. This improvement reflects the synergistic effect of VA and MW, which appears to positively influence the reaction kinetics and the polymeric structure of the matrix.

## Conclusion

The aim of this research is to produce a sustainable HS-FRCM and HS-FRGM by incorporating high contents of VA, with using MW as the mixing water. The study also investigates the impacts of four different methods of curing (tap water, seawater, sunlight, and air) on the properties of the mortars. The proposed HS-FRCM and HS-FRGM were evaluated based on several criteria, including compressive strength, uniaxial tensile strength, flexural strength, durability properties, and microstructural characteristics. The conclusions of this research are summarized as follows:

- In HS-FRCM mixtures, slump increased with higher VA content, reaching a 150% rise at 80% VA compared to the control mix. Using MW further improved workability, increasing slump by 100% in VC and 225% in VC20. In HS-FRGM mixtures, VA slightly reduced slump (up to 18%), but MW improved it, with VG and VG20 showing increases of 9% and 14%, respectively.
- Replacing PC and GGBFS with VA in HS-FRCM mixtures gradually reduced compressive strength at 40%, 60%, and 80% VA. However, 20% VA had no negative impact. When combined with MW, the compressive strength of VC20 increased by 6% at 28 days. A similar trend was observed in HS-FRGM mixtures, with generally higher strength values.
- In HS-FRCM and HS-FRGM mixes with 60% and 80% VA, compressive strengths of 29 MPa and 32 MPa were reached after 28 days of tap water curing, respectively. These values meet typical structural requirements, showing VA’s effectiveness as an alternative to traditional materials.
- The lowest compressive strength achieved in HS-FRCM and HS-FRGM mixes using VA and MW was 49 MPa. This highlights the importance of utilizing sustainable VA and MW to achieve high compressive strength without relying on traditional curing method, cementitious materials, or conventional geopolymer materials.
- Using 20% VA and MW gave flexural strengths of 8 MPa (VC20-MW) and 11 MPa (VG20-MW), similar to the control mixes. The same pattern was seen in tensile strength, showing that VA and MW help improve ductility and control cracking.
- When exposed to high temperatures of up to 300 °C or rapid freeze-thaw cycles, it can be stated that using VA up to 80%, particularly in the HS-FRGM mix, reduces the phenomena of weight loss and compressive strength loss. The use of VA and MW revealed a positive effect in decreasing the mass and compressive strength losses under both elevated temperature and rapid freeze and thaw.
- Microstructure analysis showed that VA increased C-S-H gels and lowered the Ca/Si ratio, improving strength and durability. MW reduced pores by enhancing hydration, supporting the improved mechanical properties.

## Data Availability

All data generated or analyzed during this study are included in this published article.
